# Effects of Whole-Body Electromyostimulation on Jumping, Sprinting and Agility Performance in Sportspeople and Athletes: Systematic Review and Meta-Analysis

**DOI:** 10.3390/jfmk11010033

**Published:** 2026-01-13

**Authors:** Mona Püttner, Matthias Kohl, Simon von Stengel, Andre Filipovic, Michael Uder, Wolfgang Kemmler

**Affiliations:** 1 Institute of Radiology, University Hospital Erlangen, 91054 Erlangen, Germany; mona.puettner@fau.de (M.P.); simon.von.stengeh@fau.de (S.v.S.); michael.uder@uk-erlangen.de (M.U.); 2 Faculty III: Health, Medical and Life Sciences, Black Forest University Furtwangen, 78054 Schwenningen, Germany; matthias.kohl@hs-furtwangen.de; 3 Sportclub, Verein für Bewegungsspiele (VfB) Stuttgart, 70329 Stuttgart, Germany; andre.filipovic@gmx.net

**Keywords:** electrostimulation, neuromuscular electromyostimulation, athletic performance, jump, sprint, agility

## Abstract

**Background:** Whole-body electromyostimulation (WB-EMS) is a training technology that enables the stimulation of all the main muscle groups with dedicated intensity, attracting many sportspeople and athletes of various disciplines. The aim of this systematic review and meta-analysis was to determine the effect of WB-EMS on maximum jump, sprint, and agility performance in exercising cohorts. **Methods:** Systematic literature research of five electronic databases up to March 2025, according to the Preferred Reporting Items for Systematic Reviews and Meta-Analyses (PRISMA) scheme and including interventional trials with at least one WB-EMS and one active or inactive control group that focus on maximum jump, sprint, and agility performance in sportspeople and athletes. Applying a random-effect model that includes the inverse heterogeneity model (IVhet), effects sizes (SMD), and calculates 95% confidence intervals (95%-CIs). Subgroup analyses addressed superimposed WB-EMS application vs. underlying voluntary exercise. **Results:** Twelve studies with 145 participants in the WB-EMS and 148 participants in the control group were included. Most trials on jumping (10 of 12) and all trials on sprinting and agility performance applied superimposed WB-EMS protocols compared with underlying voluntary exercise. We observed no significant positive effects of WB-EMS on maximum jump (12 studies, SMD: 0.34, 95%-CI: −0.35 to 1.03), sprint (8 studies, SMD: 0.07, 95%-CI: −0.66 to 0.80), and agility performance (5 studies, SMD: −0.11, 95%-CI: −1.28 to 1.06). Heterogeneity between the trial results was considerable (I^2^ > 80%) in all cases. **Conclusions:** Superimposed WB-EMS compared to the underlying predominately near-maximum to maximum intensity voluntary exercise provides only limited additional effects on jumping, sprinting, and ability performance.

## 1. Introduction

Due to its time-effective, joint-friendly, and highly customizable character, whole-body electromyostimulation (WB-EMS) attracts sportspeople and athletes from a large variety of disciplines [1]. Although some studies on WB-EMS application in sportspeople focus on outcomes related to regeneration or health/safety aspects, less surprisingly, the vast majority of trials address performance outcomes [1]. This may be particularly related to the time-effective and joint-friendly character of WB-EMS [2], which allows for safe and highly individualized exercise training. While WB-EMS should be considered more as a vehicle for different types of exercise protocols than a training method per se, the vast majority of trials applied WB-EMS using a resistance-type approach [1,3]. This includes less frequent, short bouts of intense intermitted stimulation (e.g., 1 × 20 min/week, 6 s of impulse–4 s impulse break). In contrast to WB-EMS in the health-related domain, most studies with sportspeople and athletes applied “superimposed WB-EMS”, a concept that combines intense volitional, predominately sport-specific exercises with simultaneous electrical stimulation. Thus, it is understandable that most longitudinal studies on WB-EMS focus on outcomes related to strength and power [1]. However, depending on the discipline, jumping, sprinting, and agility performance might be more relevant for the majority of sportspeople and athletes. In a few studies, WB-EMS trials focus on the effect of (predominately superimposed) WB-EMS (e.g., [4,5,6,7]) on jumping, sprinting, and agility performance. Although some studies reported positive outcomes on jumping and sprinting performance [5,8], most studies failed to reach statistically significant effects. This might be due to the usually limited sample sizes of exercise studies with sportspeople and athletes. In addition, most trials compare their superimposed WB-EMS approach with the underlying intense volitional exercises (without WB-EMS). This approach largely enables the isolated effect of WB-EMS within the superimposed approach to be estimated; however, due to the presumably limited additive effects, sample size and corresponding statistical power to determine significant effects should be rather high. Thus, the aim of the present systematic review and meta-analysis is to provide reliable evidence on the favorable effects of WB-EMS on jumping, sprinting, and agility performance in hobby and advanced sportspeople, as well as athletes of varying disciplines. We hypothesize that the general effect of WB-EMS, be it superimposed or not, versus non-specific controls is significant for (1) maximum jumping, (2) sprinting, and (3) agility performance. More specific hypotheses are that superimposed WB-EMS provides significant effects on (4) jumping, (5) sprinting, and (6) agility performance in comparison to the underlying volitional exercise.

## 2. Materials and Methods

The present meta-analysis is based on the systematic literature search of the comprehensive systematic review and evidence map of Reinhardt et al. [1], albeit applying more detailed eligibility criteria with respect to study outcomes (Table S1). The project closely followed the preferred reporting items for systematic reviews and meta-analyses (PRISMA) statement and was registered under PROSPERO ID CRD420250646327.

### 2.1. Eligibility Criteria

#### 2.1.1. Population

Athletes, advanced sportspeople/physical education/sport students, and recreational/hobby sportspeople who exercised at least twice weekly during the last 2 years were included. Of note, advanced sportspeople included competitive sportspeople and physical education/sport students, while semi-professional/full-professional sportspeople were categorized as “athletes” [9].

#### 2.1.2. Intervention

Only studies that applied whole-body electromyostimulation, defined as “simultaneous application of electric stimuli via at least six current channels or participation of all major muscle groups, with a current impulse effective to trigger muscular adaptations” [10], were included.

#### 2.1.3. Comparators

Studies that implemented one or more active or non-training control group were included. Studies that compared two WB-EMS protocols (e.g., [11,12]) were not considered.

#### 2.1.4. Outcomes

In contrast to the comprehensive search of the evidence map of Reinhardt et al. [1], which accepted all types of exercise and can be considered as the basis of the present study, this systematic review and meta-analysis focuses on outcomes specifically related to jumping, sprinting, and agility performance.

#### 2.1.5. Study Design

Only longitudinal controlled trials (randomized or not) were included in the present systematic review.

### 2.2. Information Sources

**Figure 1:**
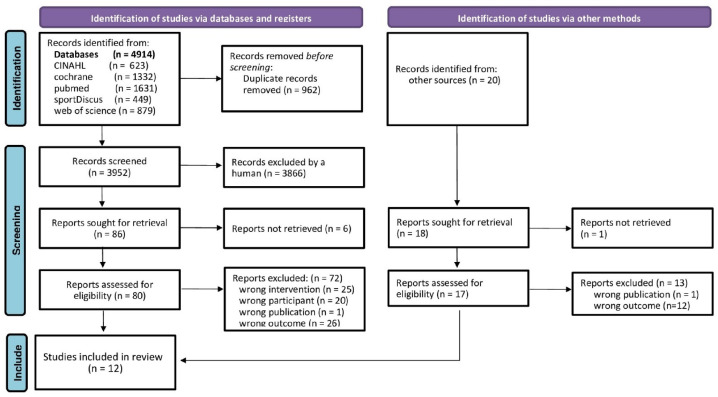
Flow chart of the present systematic literature search according to PRISMA [13].

In summary, the publications of five electronic databases (CINAHL (via Ebsco) Host, CENTRAL, Medline (PubMed), SPORTDiscus (via Ebsco Host), Web of Science (via Clarivate)), from their initiation up to 6 March 2025, were searched without language restrictions (Figure 1).

### 2.3. Literature Search

During the comprehensive search [1], a standard protocol was developed and a controlled vocabulary (MeSH term for MEDLINE, CINAHL^®^ Subject Headings for CINAHL) was applied. Keywords and their synonyms were used in the following queries: WB-EMS OR “whole body electro myo stimulation” OR electromyostimulation OR “electrical muscle stimulation” OR electro-myo-stimulation OR electrostimulation OR “integral electrical stimulation” OR “whole-body electrical muscle stimulation”) AND (athletic OR athlete OR sport OR performance OR trained). Further, reference lists of eligible articles were screened.

### 2.4. Selection Process

The selection process was based on the results of a recently published systematic literature review and evidence map [1] that applied the identical eligibility criteria except for the PICO aspect of “outcomes”. In the latter project, titles, abstracts, and full texts were independently screened against the eligibility criteria (PICOS) by three reviewers (SR, MP, and WK). Disagreements were resolved by discussion. Authors were contacted (by email) for a maximum of three times within four weeks to provide missing, incomplete, or unclear data. For the present systematic review and meta-analysis, we used the comprehensive search results of Reinhardt et al. [1] and concentrated our search on the aspect of “outcomes”.

### 2.5. Data Management

Search results were downloaded and title, abstract, and full-text screening was conducted using endnote (version 21) (Clarivate, Philadelphia, PA, USA). Duplicates were identified and excluded according to Bramer et al. [14].

### 2.6. Data Extraction

One author (MP) extracted data of the eligible studies using a Microsoft Excel table and another author (WK) checked the results. Disagreements were resolved by discussion. The sheets of the table were separated into five categories: (a) Study and publication characteristics. (b) Cohort/participant characteristics. (c) Intervention characteristics details of the WB-EMS protocol. (d) Loss to follow-up, attendance, and adverse effects. (e) Outcomes. Particular emphasis was placed on the exercise protocols of the intervention and control groups that were categorized according to superimposed WB-EMS (yes or no) and exercise of the control group (i.e., identical voluntary exercise compared to WB-EMS, newly added intervention, ongoing training routine).

### 2.7. Quality Assessment

Using the physiotherapy evidence database (PEDro) scale risk of bias tool [15], two independent reviewers (MP and WK) assessed the studies for risk of bias. Disagreements were resolved by discussion. Methodological quality of the studies was classified as follows: <5 score points: low; 5–7 score points: moderate; >7 score points: high [16].

### 2.8. Data Synthesis

Briefly, where applicable missing standard deviations (SDs) were converted from standard errors (SEs) and confidence intervals (CIs) [17]. When measures for variation in the change were not reported, we imputed the (mean) SD by using the correlations between baseline and final values from the other studies [17].

Study, publication, cohort, participant, and intervention characteristics are shown in tables. Core study outcomes included in the analysis were jumping performance as determined by counter movement/vertical jumps, sprint performance as assessed by linear sprinting, and agility as determined by multiple direction sprints.

### 2.9. Statistical Analysis

Random-effects meta-analyses were calculated using R statistical software (4.5.1 patched [18]) and the metafor package [19]. Continuous outcome data were synthesized using standardized mean differences (SMDs) with 95% confidence intervals (95% CIs). We performed a meta-analysis, applying the robust inverse heterogeneity (IVhet) model [20] as the primary method of analysis. The Cochran Q test and the I^2^ statistic determined heterogeneity between the trials results. I^2^ values of 0–40% were considered as “low”, 30–60% as “moderate”, 50–90% as “substantial”, and 75–100% as considerable heterogeneity [21]. Apart from traditional funnel plots, regression and rank correlation effect estimates, and their standard errors, we also applied a trim-and-fill analysis using the L0 estimator, as proposed by Duval et al. [22]. Further, we checked for asymmetry using Doi plots and the Luis Furuya-Kanamori index (LFK index) [23]. LFK values of ±1 to ±2 were considered as indicators of minor asymmetry; meanwhile, LFK indices higher than ±2 suggested major asymmetry. Sensitivity analyses were applied to determine whether the overall result of the analysis is robust to the use of the imputed correlation coefficient (minimum or maximum SD). As stated, for the main analysis we used the result obtained by applying the mean SD of the correlations.

## 3. Results

Of the 4914 records in total, 12 randomized controlled trials with 13 group comparisons [4,5,6,7,8,24,25,26,27,28,29,30] were included in the present systematic review (Figure 1).

### 3.1. Publication and Study Characteristics

Table 1 displays publication and study characteristics of the included projects. Apart from one non-randomized controlled trial [26], all the others were randomized controlled trials with parallel group designs or randomized controlled cross-over design [29]. All studies compared a single WB-EMS group with a single control group. Studies were published between 2016 and 2022, by research groups from Germany (n = 6; [4,5,6,7,8,29]), Spain (n = 3, [24,28,30]), Turkey (n = 2, [25,27]), and Iraq [26]. The number of participants per group ranged between n = 5 [26] and n = 26 [29] sportspeople.

### 3.2. Participant Characteristic

Table 1 shows the participant characteristics of the included studies. Apart from one study on rehabilitation [26], all the studies included healthy sportspeople and athletes. Two studies focused on hobby sportspeople [24,30], seven studies included advanced sportspeople [4,6,7,8,27,28,29], and three projects included semi-professional/professional athletes [5,25,26]. Some participants of the studies had a team sports (n = 4) and running (n = 1) background, while seven studies focused on allrounders (including sport/physical education studies) (Table 1). Eight studies included only males, two studies only females, and the remaining two studies included mixed cohorts (Table 1). Participants of the included studies were aged on average between 20 and 30 years; however, one study included younger men 15–20 years old. When applying a BMI-based cut-off, participants of the studies can be considered to be of normal weight (Table 1).

### 3.3. Exercise Characteristic

Table 2 summarizes the exercise protocols of the studies. Most important for the present work, most studies (11 of 12) prescribed superimposed WB-EMS protocols and (10 of 11 studies) compared superimposed WB-EMS with the underlying volitional exercise. The latter included static (resistance) exercise, DRT, jumps/plyometric exercise, cycling, and running (Table 2). Duration of the trials are usually short, varying from 4 [8] to 14 weeks [5]. Weekly WB-EMS volume varied considerably between 13 min (1 × 13 min/week, [28]) to a rather unusual 3.5 h/week (3.5 × 60 min [8]), while most projects applied protocols with 1–2 sessions of 9–25 min/session per week (Table 2). All projects applied bipolar (biphasic) stimulation in the low-frequency range [2] (Table 2). In parallel, the studies used an impulse breath of 350 or 400 µs. All the studies used intermittent WB-EMS protocols with short bouts of stimulation (usually during the voluntary exercise) with short impulse breaks (Table 2). Due to the superimposed WB-EMS protocol of most of the studies, it is difficult to report exercise or impulse intensity separately since exercise intensity is derived from the combination of voluntary exercise and WB-EMS application. Nevertheless, although difficult to rate in some cases (e.g., “60–100% device capacity”), we conclude that (after a short conditioning period) all the studies provided at least moderate to high exercise intensity, with two authors [8,28] reporting maximum tolerable intensity (Table 2). Hereby it is important to note that it must remain possible to perform the sport-specific movement correctly.

### 3.4. Control Group Characteristics

As stated, ten of the twelve control groups applied the same voluntary exercises as the (superimposed) WB-EMS groups and thus allow the isolated effect of WB-EMS during superimposed application to be determined (Table 2). One project [24] compared DRT and high-intensity running exercise superimposed by WB-EMS with the ongoing routine. The only non-superimposed WB-EMS study [26] compared WB-EMS intervention with a rehabilitation program that focused on DRT.

### 3.5. Adverse Effects, Loss to Follow-Up, Attendance (WB-EMS Group)

Three studies did not provide information on adverse effects, and the remaining ten studies did not report unintended side effects related to WB-EMS application (Table 2). Nevertheless, 24 h after superimposed WB-EMS, Filipovic et al. [5] observed moderately increased creatine kinase (CK) levels. This is a finding that is reported particularly after early, intensive WB-EMS application, but which has no clinical consequences, as is typical for exertional rhabdomyolysis [32]. Loss to follow-up/withdrawal rate was provided by 10 studies (Table 2). In four studies no participants were lost or withdrawn, and in the remaining studies the loss to follow-up rate averaged between 15% and 21%. In summary, reasons for loss to follow-up were predominately due to illness or injuries not related to the interventions. Owing to the offer to make up missed sessions, the 10 studies on this issue reported attendance rates for the WB-EMS groups of around 100% (Table 2).

### 3.6. Methodologic Quality

**Figure 2:**
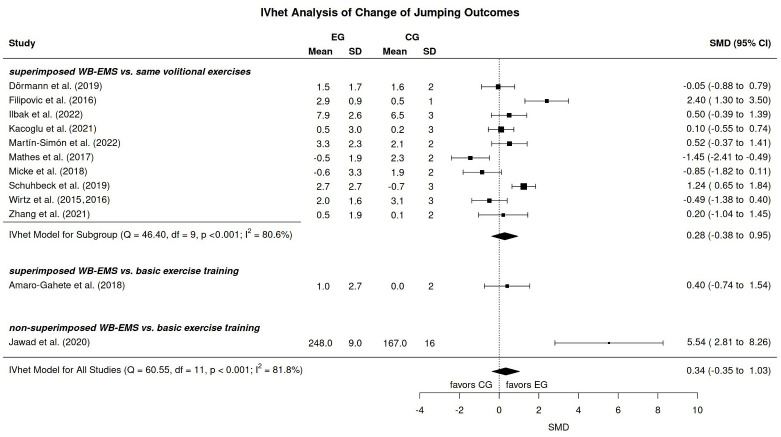
Forest plot WB-EMS effects for maximum jump performance [4,5,6,7,8,24,25,26,27,28,29,30,31].

Methodologic quality according to PEDro [15] is shown in Table 1. In summary, the methodological quality ranged from 3 to 5 out of a maximum of 10 PEDro score points and can be thus considered as low-to-moderate at best [16]. The reasons for this finding predominately relate to issues related to “allocation concealment”, “blinding of all subjects”, “blinding of all therapists”, “blinding of all assessors”, and/or “explicit statement that all subjects received treatment or control conditions as allocated”. Furthermore, for three studies that failed to provide loss to follow-up (Figure 2), the PEDro criterium of “key outcome measures from more than 85% of the subjects” was not fulfilled. Bearing in mind that blinding of subjects and therapists is not reliably applicable in exercise trials, eight PEDro score points should be considered as a realistic maximum for WB-EMS trials.

### 3.7. Outcomes

All 12 projects reported outcomes related to jumping performance (Table 3). Apart from one study that reported maximum vertical jumping performance in Newton (N) [26], all the other projects listed maximum jumping height in m/cm. Eight projects reported results related to sprinting performance (in s) [4,5,6,7,25,27,28,29] (Table 3). Apart from one study [29] that determined (ice-hockey) skating performance, all the other studies focused on running. Lastly, five studies [4,5,6,7,25] addressed agility performance as determined by sprinting (running) exercises (in s) (Table 3).

### 3.8. Meta-Analysis Results

#### 3.8.1. Jumping

In summary, independently of imputed mean (primary analysis), minimum, or maximum SD, we did not observe significant (*p* = 0.34) effects of WB-EMS vs. unspecific control on jumping performance (SMD: 0.34, 95%-CI: −0.35 to 1.03) in sportspeople and athletes (Figure 2). A largely similar result (SMD: 0.28, 95%-CI: −0.38 to 0.95) was observed for the 10 (of 12) trials that focused on the comparison of superimposed WB-EMS versus volitional exercise alone. Also, as expected both analyses revealed a similarly substantial heterogeneity between the trial results. Thus, we have to reject hypotheses 1 and 4 that suggested significantly more favorable changes among the WB-EMS group.

##### Publication/Small Study Bias “Maximum Jump Performance”

**Figure 3:**
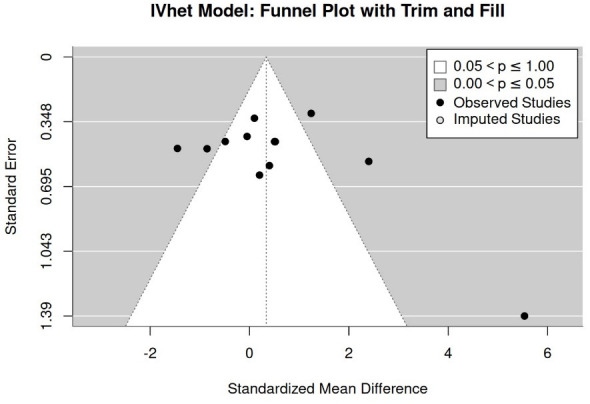
Funnel plot of study results for maximum jumping performance.

We included all the studies regardless of the category recorded from the analyses for publication/small study bias/asymmetries. Firstly, a funnel plot with trim-and-fill analysis suggests no evidence for a publication/small study bias (Figure 3). In parallel, rank (*p* = 0.74) and regression (*p* = 0.53) tests for funnel plot asymmetry did not reveal significant results. Finally, the LFK index (1.08) shows only minor asymmetry.

#### 3.8.2. Sprinting Performance

**Figure 4:**
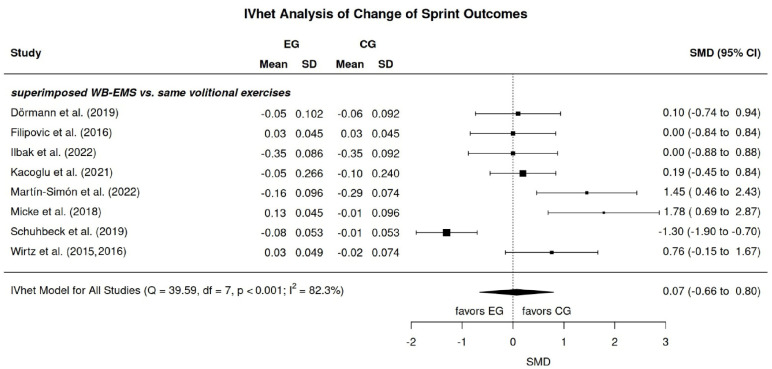
Forest plot WB-EMS effects for sprint performance [4,5,6,7,25,27,28,29,31].

All studies focusing on sprinting performance in athletes and sportspeople applied superimposed WB-EMS protocols that were compared with the underlying volitional exercise. In short, independently of the imputation strategy (mean, minimum, or maximum SD), we observed no effect of WB-EMS application on sprint performance (SMD: 0.07, 95%-CI: −0.66 to 0.80) (Figure 4). Again, heterogeneity of the trial results was substantial, with two studies reporting pronounced negative effects [6,28] and one study [29] reporting significant positive effects. Consequently, we must reject hypotheses 5 and 2, which anticipate a significant advantage for the WB-EMS approach.

##### Publication/Small Study Bias “Maximum Sprinting Performance”

**Figure 5:**
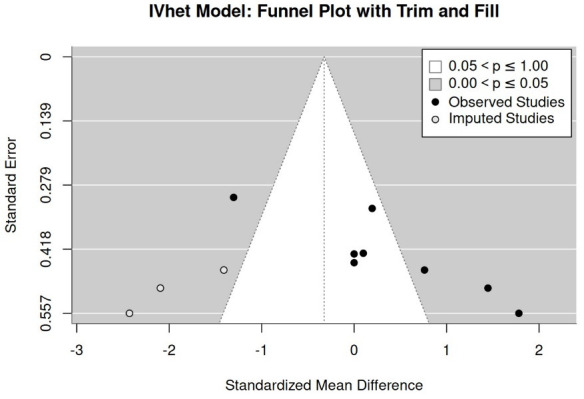
Funnel plot of study results for sprint performance.

The funnel plot indicates significant asymmetry (Figure 5). Imputing the estimated number of missing studies (n = 3) on the left side (favors WB-EMS) however did not result in significant results (SMD: 0.40, 95%-CI: −1.10 to 0.45). Rank (*p* = 0.03) and regression tests (*p* = 0.008) for funnel plot asymmetry confirmed the significant asymmetry; however, the LFK index suggests only minor asymmetry (1.37).

#### 3.8.3. Agility Performance

**Figure 6:**
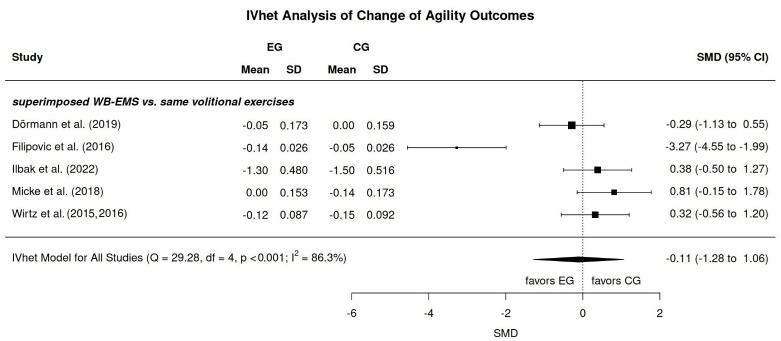
Forest plot WB-EMS effects for agility performance [4,5,6,7,25,31].

In parallel to sprint outcomes, all five WB-EMS trials in the area of agility emphasize the comparison of superimposed WB-EMS versus underlying voluntary exercise (Figure 6). Briefly, independently of the imputation strategy no relevant effects of superimposed WB-EMS on agility performance were determined (SMD: −0.11, 95%-CI: −1.28 to 1.06). Substantial heterogeneity can be observed between the trial results. In summary, hypothesis 6 (and 3 correspondingly), i.e., WB-EMS training contributes favorable effects to agility performance among athletes and sportspeople, must be rejected.

##### Publication/Small Study Bias “Agility Performance”

**Figure 7:**
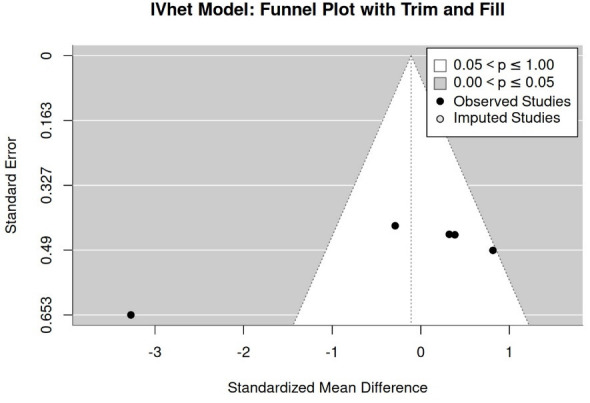
Funnel plot of study results for agility performance.

The funnel plot with trim-and-fill analysis (Figure 7), as well as rank (*p* = 0.81) and regression tests (*p* = 0.10) for funnel plot asymmetry, provided no evidence for a publication/small study bias (Figure 5) for agility performance. Nevertheless, the LFK index (1.017) shows minor asymmetry. However, due to the low number of studies, results should be interpreted with care.

## 4. Discussion

In the present systematic review and meta-analysis that aimed to summarize and quantify the effects of WB-EMS on maximum jumping, sprinting, and agility performance in sportspeople of different performance levels (i.e., hobby sportspersons to professional athletes) we failed to determine significant effects. Several aspects might contribute to this negative result. Firstly, most of the studies focusing on maximum jumping performance, as well as all of the studies emphasizing maximum sprinting and agility performance, applied superimposed WB-EMS, which was then compared with the underlying voluntary exercises. Although the intensity of voluntary exercise is not always reported in full, it appears that in many cases it was performed at a near-maximum to maximum intensity (e.g., [5]). In other words, superimposed WB-EMS is merely the “icing on the cake” and might therefore only provide a limited contribution to the overall result. Although this sounds logical, Filipovic et al. [5], who specified only 3 × 10 maximal squat jumps, reported significantly positive results of added WB-EMS on maximum jump performance in their cohort of professional soccer players. In contrast, Micke et al. [6], who applied their jumping sequence with near-maximum to maximum voluntary intensity, detected significantly more favorable effects on CMJ (however, not squat jump, drop jump, or standing long jump) in their control group (Figure 2). Also surprisingly, Mathes et al. [8], who compared the effects of 60 min of cycling at 60% peak power output (PPO) with and without superimposed WB-EMS, determined significant improvement of CMJ performance (not squat and drop jump) in the CG (*p* = 0.01 vs. WB-EMS). Although it is rather unlikely that the additional WB-EMS decreases the effects of voluntary exercise, we are unable to explain this unexpected finding. It is also worth noting that most authors [4,6,8,24,27] who conducted multiple jump tests found that the CMJ test showed the lowest effect of WB-EMS (whereas the opposite effect has never been reported). However, is there any rational explanation for a decreased sensitivity of the CMJ to whole-body EMS? Briefly reviewing the results of the two studies that addressed the effects of superimposed [24] or non-superimposed WB-EMS [26] versus the habitual exercise routine on maximum jumping performance result also fails to yield a clear result. Amaro-Gahete et al. [24], who replaced one of two running sessions/week with high-intensity DRT, power, and interval running superimposed with WB-EMS, reported largely similar effects on CMJ compared to the ongoing training routine. On the other hand, Jawad et al. [26], who supplemented non-superimposed WB-EMS as part of their rehabilitation program for hip injuries in professional footballers, observed very pronounced WB-EMS effects on vertical jump performance (Figure 2). In summary, although some trials provided evidence of WB-EMS-induced effects on maximum jumping power, the factors underlying the success are unclear, particularly due to pronounced differences in training programs and partially incomplete reporting of exercise training details.

While it could be speculated that only random effects prevented a more positive result for the effects of WB-EMS on maximum jumping performance, this is far less the case for maximum sprint and agility performance. Although all the trials specified strength- and/or power-related voluntary exercises superimposed by widely comparable WB-EMS protocols, heterogeneity between the trial results was “considerable” (≥80%), with in essence negligible effects compared to voluntary exercise alone. Reviewing the individual articles for the underlying reasons of these findings is a daunting task. With regard to maximum sprint performance, the question arises as to how can it be that a control group which performed either “traditional” plyometric exercises [28] or a mixed strength and jumping protocol [6] achieved significantly higher improvements than a group with the same exercises but superimposed with WB-EMS. In diametral contrast, Schuhbeck et al. [29], who in effect applied a jumping and static, dynamic and isokinetic resistance exercise training superimposed (vs. not) by WB-EMS, i.e., a training regime that did not significantly differ from the exercise regimes above, reported very pronounced positive effects on linear (ice hockey) sprinting performance.

Reviewing the literature for neuromuscular EMS effects on performance parameters, two meta-analyses [33,34], which included predominately local EMS application, confirmed a large heterogeneity of trial results which is dependent on the underlying or additive voluntary exercise. Although not fully applicable to the present study and of limited statistical power, Micke et al. [34] reported the most favorable effects for (compared to an active control group) DRT superimposed by EMS with additional jumping, jump training superimposed by EMS, and body weight DRT superimposed by EMS.

Some study limitations and particularities of this systematic review should be addressed to allow the reader to properly interpret the results. (1) Although we focus on hip/lower extremity strength and power, we do not include trials that applied local EMS. This decision is based on the larger size of the WB-EMS cuff electrodes that stimulate the entire thigh or calf region, and also on the aspect that gluteal and trunk muscles, i.e., muscle groups involved in leg press or squatting exercises, were included. (2) There is currently no reliable definition of “sportspeople”. Thus, we applied the criterion of ≥2 sessions/week during the last 2 years to categorize “hobby sportspeople”. Accordingly, physical education students and sportspeople who participate in competitions were classified as “advanced sportspeople”, while semi-professional and professionals were considered as “athletes” [9]. Hence, our cohort of sportspeople cannot be considered as a homogeneous group, although the high relevance of changes in performance outcomes is a shared characteristic. Nevertheless, due to the limited statistical significance, we refrained from determining differences in performance development between the groups. (3) We accepted both non-superimposed and superimposed WB-EMS protocols [4,5,6,7,8,27,28,29,30,31,35,36,37,38,39,40,41], fully aware that the effect of superimposed WB-EMS on a given outcome is provided by both voluntary and electrical muscle stimulation. (4) We aimed to adequately consider exercise characteristics of the control groups appropriately so as to appraise the study results. We categorized control groups into groups that (a) performed the similar voluntary exercise as the WB-EMS-group (e.g., [4,5]), (b) added new exercise interventions (e.g., [35]), or (c) maintained their regular training routine (e.g., [40]). Results should be interpreted depending on the scope of application. Therefore, if the interest lies in the isolated effect of superimposed WB-EMS, the corresponding protocols must be compared with control groups that perform identical voluntary exercises. However, when considering superimposed WB-EMS as a whole, it is justified to compare it with control groups that maintain their exercise routine. (5) We applied the PEDro scale to rate methodological quality (Table 1). While PEDro [15] is specifically designed for physiotherapy and exercise studies, overall certainty of the body of evidence was not addressed by this score. (6) It is difficult to summarize the external validity of our results. Although we focused primarily on a sporting collective, we did not examine any differences between cohorts with different statuses. Further, the vast majority of trials applied superimposed WB-EMS, a training method less frequently used in non-athletic cohorts [3,42]. Taking these limitations into account, we nevertheless would like to generalize the results to sportive cohorts looking to superimpose WB-EMS on their conventional voluntary exercise.

## 5. Conclusions

In general, superimposed WB-EMS provides only limited additional effects on jump, sprint, and ability performance—at least when compared with predominately near-maximum to maximum intensity voluntary exercise. However, considering that even small improvements in performance are important for sportspeople and particular athletes, the sample size and statistical power might be still too limited to determine clinically meaningful changes. Well-designed WB-EMS trials with adequate sample sizes should address this issue to clearly determine the relevance of superimposed WB-EMS in exercising cohorts.

## Figures and Tables

**Table 1:** Study and participant characteristics of the studies.

	Author	Publication Year	Study Design	Study Groups/ Conditions [n]	Total Sample Size [n]	Gender	Age [Years]	BMI [kg/m^2^] ^1^	Status	Discipline	Methodological Quality (PEDro)
1	Amaro-Gahete et al. [24]	2018	RCT	2	14	m	27 ± 7	23 ± 3	Hobby sportspeople	Runners	4
2	Dörmann et al. [4]	2019	RCT	2	28	w	21 ± 2	22 ± 2	Advanced sportspeople	Allrounders	4
3	Filipovic et al. [5]	2016	RCT	2	22	m	26 ± 3	24 ± 2	Semi-/full-professionals	Soccer players	4
4	Ilbak et al. [25]	2022	RCT	2	20	m	15–20	22	Semi-/full-professionals	Basketball players	4
5	Jawad et al. [26]	2020	RCT	2	10	m	ng	ng	Semi-/full-professionals	Soccer players	3
6	Kacoglu et al. [27]	2021	RCT	2	38	m + w	22 ± 3	22 ± 2	Advanced sportspeople	Allrounders	4
7	Martín-Simón et al. [28]	2022	RCT	2	20	m + w	19–25	23	Advanced sportspeople	Allrounders	4
8	Mathes et al. [8]	2017	RCT	2	24	m	23 ± 5	23	Advanced sportspeople	Allrounders	5
9	Micke et al. [6]	2018	RCT	2	18	m	23 ± 3	22 ± 2	Advanced sportspeople	Allrounders	5
10	Schuhbeck et al. [29]	2019	RCOT	2	30	m	28 ± 8	24	Advanced sportspeople	Ice-hockey players	5
11	Wirtz et al. [7,31]	2015, 2016	RCT	2	20	m	22 ± 2	24	Advanced sportspeople	Allrounders	4
12	Zhang et al. [30]	2021	RCT	2	10	w	27 ± 4	22	Hobby sportspeople	Resistance exercise	4

BMI: body mass index; ng: not given; PEDro: physiotherapy evidence database scale risk of bias tool; RCT: randomized controlled trial; RCOT: randomized cross-over trial. ^1^: If not specified, BMI was calculated based on body height and body mass (see BMI data without SD).

**Table 2:** Exercise characteristics of the included studies.

	Author	Study Length [Weeks]	Superimposed Exercise?	Comparable Voluntary Exercise in Control Group?	EMS-Sessions n/Week × Length [min]	Exercise/WB-EMS Protocol Impulse Frequency [Hz], -Width [µs], -Duration [s], -Break [s], -Intensity [RPE] (Additionally to Sport-Specific Exercise)	Exercise/Activity in the Control Group(s) (Without WB-EMS) Additional to Sport-Specific Exercise)	Loss to FU [%]/ Attendance [%]/ Adverse Effects
1	Amaro-Gahete et al. [24]	6	yes	no	1 × 12–20	High intensity DRT, power and (interval) running superimposed by WB-EMS: Variable, undulated periodized WB-EMS: 12 and 90 Hz, 350 µs, 4–30 s, 4–30 s, 10–17 [CR 20]	Regular running routine only (2 sessions/week)	14/96/no
2	Dörmann et al. [4]	4	yes	yes	2 × 20	DRT (see control) superimposed by WB-EMS, 85 Hz, 350 µs, impulse during exercises, RPE ≥16 (CR20)	DRT: 4 ex., 3 × 8–10 reps RPE ≥ 16 Power: 5 ex., 3 × 5–10 reps/3 × 8 s	21/100/no
3	Filipovic et al. [5]	14	yes	yes	2 × 9	Squat jumps: 3 × 10 reps superimposed by WB-EMS 80 Hz, 350 µs, 4 s–10 s, up to RPE 18–19 (CR20)	Squat jumps (3 × 10 reps) only	0/100/no
4	Ilbak et al. [25]	12	yes	yes	2 × 20	Plyometric jumping exercises (8 ex., 3 × 10–12 reps) superimposed by WB-EMS: 20 Hz, 350 µs, 10 s–10 s, 50–80% maximum tolerable intensity	Plyometric jumping exercises (8 ex., 3 × 10–12 reps) only	0/100/no
5	Jawad et al. [26]	8	no	no	3 × 20	WB-EMS: 85 Hz, 350 µs, continuous impulse, RPE 6–8 (CR10)	Rehabilitation program (19 DRT exercise, 3–4 × 10–20 reps) only	ng
6	Kacoglu et al. [27]	6–4	yes	yes	2 × 25	DRT: seated leg press (3 × 20 reps) superimposed by WB-EMS: 100 Hz, 400 µs, 5 s–10 s, RPE 8–9 (CR 10)	DRT: seated leg press (3 × 20 reps) only	ng
7	Martín-Simón et al. [28]	6	yes	yes	1 × 13	3 sessions, 100–140 jumps with 1 session superimposed by WB-EMS: 120 Hz, 350 µs, 5 s–10 s, max. tolerable intensity	Jumping (3 sessions, 100–140 jumps) only	ng
8	Mathes et al. [8]	4	yes	yes	3.5 × 60	Cycling at 60% peak power output, superimposed by WB-EMS: 80 Hz, 400 µs, 10 s–2 s, maximum tolerable intensity	Cycling (60% peak power output) only	13/100/no
9	Micke et al. [6]	8	yes	yes	2 × ≈25	DRT: 5 ex., 3 × 5−10 reps, RPE > 16 (CR20) superimposed by WB-EMS: 85 Hz, 350 µs, adjusted to exercises 70% max. intensity	DRT (5 ex., 3 × 5−10 reps) only	0/100/no
10	Schuhbeck et al. [29]	12	yes	yes	1 × 20	6 weeks of static, 6 weeks of dynamic RT exercise superimposed by WB-EMS 85 Hz, 350 µs, 4 s–4 s, ≥75% max. intensity	Static RT, DRT only	13/100/no
11	Wirtz et al. [7,31]	6	yes	yes	2 × 10	Back half squats, 4 × 10 reps to RM, superimposed by WB-EMS, 85 Hz, 350 µs, 5 s–1 s at 70% max. tolerable intensity	Back squats (4 × 10 reps to RM) only	0/100/no
12	Zhang et al. [30]	6	yes	yes	2 × 20−25	DRT: 4 exercises, 5 × to nRM at 85% 1 RM superimposed by WB-EMS 85 Hz, 350 µs, EMS during sets, 60–100% device capacity	DRT (4 exercises, 5× to nRM at 85% 1 RM) only	17/100/no

CR10: category ratio scale 10 (1–10); CR20: category ratio scale 20 (6–20); DRT: dynamic resistance exercise; FU: follow-up; ng: not given; nRM: non-repetition maximum; reps: repetitions; RPE: rate of perceived exertion; RT: resistance exercise.

**Table 3:** Strength- and power-related outcomes reported by the included studies.

	Author	Jumping Outcomes (cm)	Sprinting Outcomes (s)	Agility Outcomes (s)
1	Amaro-Gahete et al. [24]	CMJ, ABJ	----------	----------
2	Dörmann et al. [4]	SLM, CMJ, SJ	5, 10, 20, 30 m Sprint	30 m T-run test
3	Filipovic et al. [5]	SJ, CMJ, DJ	30 m Sprint	15 m direction change test
4	Ilbak et al. [25]	CMJ (jump and reach)	20 m Sprint	Illinois agility test (≈70 m)
5	Jawad et al. [26]	Vertical jump (N)	----------	----------
6	Kacoglu et al. [27]	SJ, CMJ	40 m Sprint	----------
7	Martín-Simón et al. [28]	CMJ	20 m Sprint	----------
8	Mathes et al. [8]	SJ, CMJ, DJ	----------	----------
9	Micke et al. [6]	SJ, CMJ, DJ, SLJ	30 m Sprint	30 m Pendulum test
10	Schuhbeck et al. [29]	CMJ	10 m Skate Sprint	----------
11	Wirtz et al. [7,31]	SJ, CMJ	30 m Sprint	30 m Pendulum test
12	Zhang et al. [30]	SJ, CMJ, ABJ	----------	----------

ABJ: Abalakov jump; CMJ: counter movement jump; DJ: drop jump; SLJ: standing long jump; SJ: squat jump.

## Data Availability

No new data were created or analyzed in this study. Data sharing is not applicable to this article.

## Supplementary Materials

The following supporting information can be downloaded at: https://www.mdpi.com/article/10.3390/jfmk11010033/s1. Table S1: Underlying search strategies and their results [1].

## Abbreviations

The following abbreviations are used in this manuscript:

1 RM	One Repetition Maximum
ABJ	Abalakov Jump
CMJ	Counter Movement Jump
DJ	Drop Jump
DRT	Dynamic Resistance Exercise Training
IVhet	Inverse Heterogeneity Model
nRM	Non-Repetition Maximum
PEDro	Physiotherapy Evidence Database
Reps	Repetitions
RPE	Rate of Perceived Exertion
RT	Resistance Exercise Training
SLJ	Standing Long Jump
SMD	Standardized Mean Differences
SQ	Squat Jump
WB-EMS	Whole-Body Electromyostimulation
