# Supplementary material for: Effects of Whole-Body Electromyostimulation on Jumping, Sprinting and Agility Performance in Sportspeople and Athletes: Systematic Review and Meta-Analysis
Source: J Funct Morphol Kinesiol. 2026 Jan 13;11(1):33. doi: 10.3390/jfmk11010033 (PMC12821516; doi:10.3390/jfmk11010033)
Supplement: Supplementary file 1 [file jfmk-11-00033-s001.zip › jfmk-4061651-supplementary.pdf]

**Table S1:** Underlying search strategies and their results [1]

| Database | Search date | Search terms | Number of hits |
|---|---|---|---|
| PubMed | 21th February 2025 | (WB-EMS OR "whole body electro myo stimulation" OR electromyostimulation OR "electrical muscle stimulation" OR electro-myo-stimulation OR electrostimulation OR "integral electrical stimulation" OR "whole-body electrical muscle stimulation") AND (athletic OR athlete OR sport OR performance OR trained) | 1631 |
| Cochrane | 21th February 2025 | (WB-EMS OR "whole body electro myo stimulation" OR elektromyostimulation OR "electrical muscle stimulation" OR electro-myo-stimulation OR electrostimulation OR "integral electrical stimulation" OR "whole-body electrical muscle stimulation") AND (athletic OR athlete OR sport OR performance OR trained) | 1332 |
| CINAHL | 21th February 2025 | (WB-EMS OR "whole body electro myo stimulation" OR electromyostimulation OR "electrical muscle stimulation" OR electro-myo-stimulation OR electrostimulation OR "integral electrical stimulation" OR "whole-body electrical muscle stimulation") AND (athletic OR athlete OR sport OR performance OR trained) | 623 |
| SPORTDiscus | 21th February 2025 | (WB-EMS OR "whole body electro myo stimulation" OR electromyostimulation OR "electrical muscle stimulation" OR electro-myo-stimulation OR electrostimulation OR "integral electrical stimulation" OR "whole-body electrical muscle stimulation") AND (athletic OR athlete OR sport OR performance OR trained) | 449 |
| Web of science | 21th February 2025 | (WB-EMS OR "whole body electro myo stimulation" OR electromyostimulation OR "electrical muscle stimulation" OR electro-myo-stimulation" OR "whole-body electrical muscle stimulation") AND (athletic OR athlete OR sport OR trained) | 879 |
| Other sources | 21th February 2025 | „WB-EMS, whole body electro myo stimulation, electromyostimulation, electrical muscle stimulation, electro-myo-stimulation, electrostimulation, whole-body electrical muscle stimulation" AND „athletic, athlete, sport, trained" | 20 |
